# The ancient sarcomeric myosins found in specialized muscles

**DOI:** 10.1186/s13395-019-0192-3

**Published:** 2019-03-05

**Authors:** Lindsey A. Lee, Anastasia Karabina, Lindsey J. Broadwell, Leslie A. Leinwand

**Affiliations:** 1 Department of Molecular, Cellular, and Developmental Biology, University of Colorado, Boulder, CO USA; 2 BioFrontiers Institute, University of Colorado, Boulder, CO USA; 3 Department of Biochemistry, University of Colorado, Boulder, CO USA

**Keywords:** Ancient myosins, MYH7b, MYH15, MYH16, Extraocular muscle, Muscle spindles, Masticatory muscle

## Abstract

Striated muscles express an array of sarcomeric myosin motors that are tuned to accomplish specific tasks. Each myosin isoform found in muscle fibers confers unique contractile properties to the fiber in order to meet the demands of the muscle. The sarcomeric myosin heavy chain (MYH) genes expressed in the major cardiac and skeletal muscles have been studied for decades. However, three ancient myosins, *MYH7b*, *MYH15*, and *MYH16*, remained uncharacterized due to their unique expression patterns in common mammalian model organisms and due to their relatively recent discovery in these genomes. This article reviews the literature surrounding these three ancient sarcomeric myosins and the specialized muscles in which they are expressed. Further study of these ancient myosins and how they contribute to the functions of the specialized muscles may provide novel insight into the history of striated muscle evolution.

## Background

**Fig. 1:**
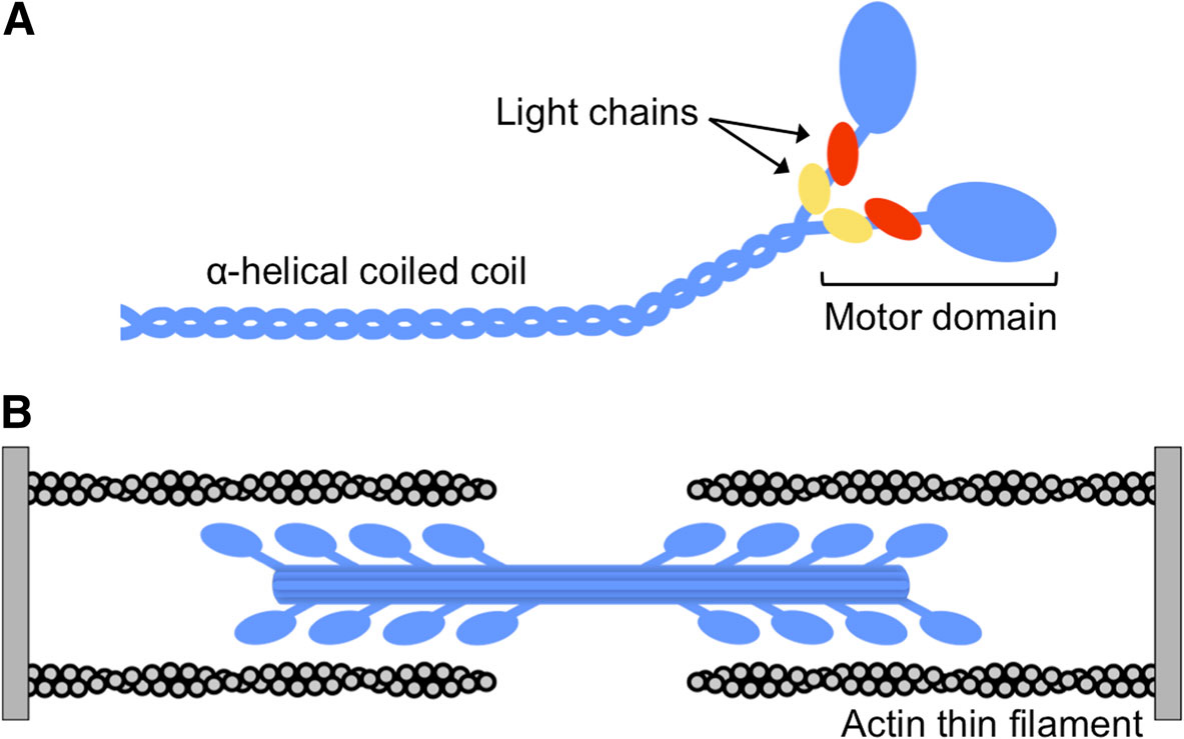
**a** Sarcomeric myosins are hetero-hexameric complexes composed of two heavy chains (blue) that are each bound by two non-identical light chains (essential light chain, red and regulatory light chain, yellow). The myosin heavy chain is distinguished by two functional units, a motor domain that contains the catalytic ATPase site and binds actin and an α-helical coiled-coil rod domain that dimerizes and assembles into biopolar thick filaments. **b** Within the sarcomere, the myosin heavy chain rod drives assembly of biopolar thick filaments (blue) where the myosin motor domains protrude off the surface to interact with the actin thin filament (gray)

Striated muscles are comprised of many heterogeneous and highly specialized fibers. Skeletal muscle fibers are extremely adaptable and can meet the varying demands of muscles by responding to changes in environmental cues in several ways. These muscle fibers can remodel their structure and contractile properties, largely by reprogramming the gene expression profiles of sarcomeric components including myosin motor proteins. Myosin produces the force necessary for a variety of cellular movements by hydrolyzing ATP and interacting with actin filaments. The myosin superfamily encodes 18 distinct classes of myosin motors, which are found ubiquitously in eukaryotes and participate in a variety of cellular motile processes (see Hartman and Spudich [1] for review). A subset of class II myosins, those that power muscle contraction in striated muscles, will be the focus of this review. The class II myosin heavy chain (MyHC) protein is comprised of two functional units, a globular motor domain that contains the catalytic ATPase site and binds actin and an α-helical coiled-coil rod domain that dimerizes and assembles into bipolar thick filaments (Fig. 1a). Striated muscle has a highly organized ultrastructure, consisting of a repeating contractile unit called the sarcomere. Within the sarcomere, myosins assemble into bipolar thick filament structures where the myosin motor domains protrude off the surface to interact with neighboring actin filaments (Fig. 1b). Myosin thick filaments and actin thin filaments interdigitate and slide past one another to cause sarcomere shortening in a concerted motion resulting in muscle contraction. This review describes the most ancient sarcomeric myosins, which have largely escaped characterization due to their unique expression patterns, variable expression levels across species, and relatively recent annotation, and provides an overview of the specialized skeletal muscles in which they function.

### Sarcomeric myosin genes

**Fig. 2:**
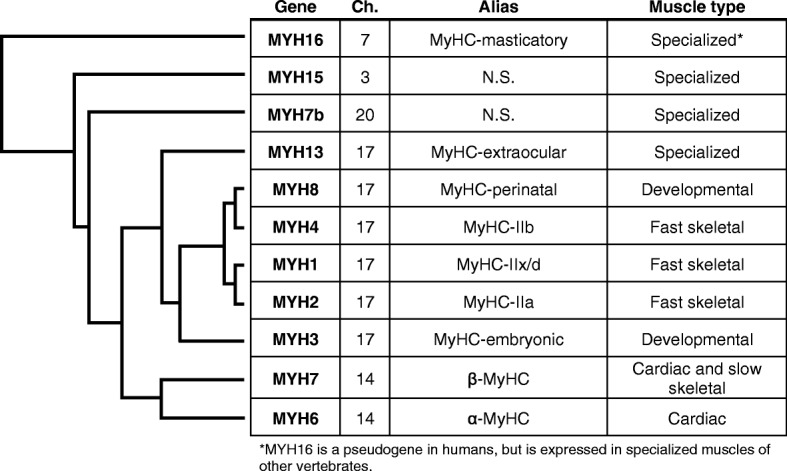
Sarcomeric myosin heavy chain genes, human chromosome (Ch.) location, protein alias (N.S. refers to no specification), and primary muscle tissue of expression are listed in the table. The phylogeny on the left indicates the evolutionary relationship between the human sarcomeric myosin heavy chain genes (this cladogram does not reflect accurate scale). Cladogram adapted with permission of the publisher from Stedman et al. 2004, copyright ©2004, Springer Nature. Scheme adapted with permission of the publisher from Rossi et al. 2010, copyright ©2010 John Wiley and Sons

**Fig. 3:**
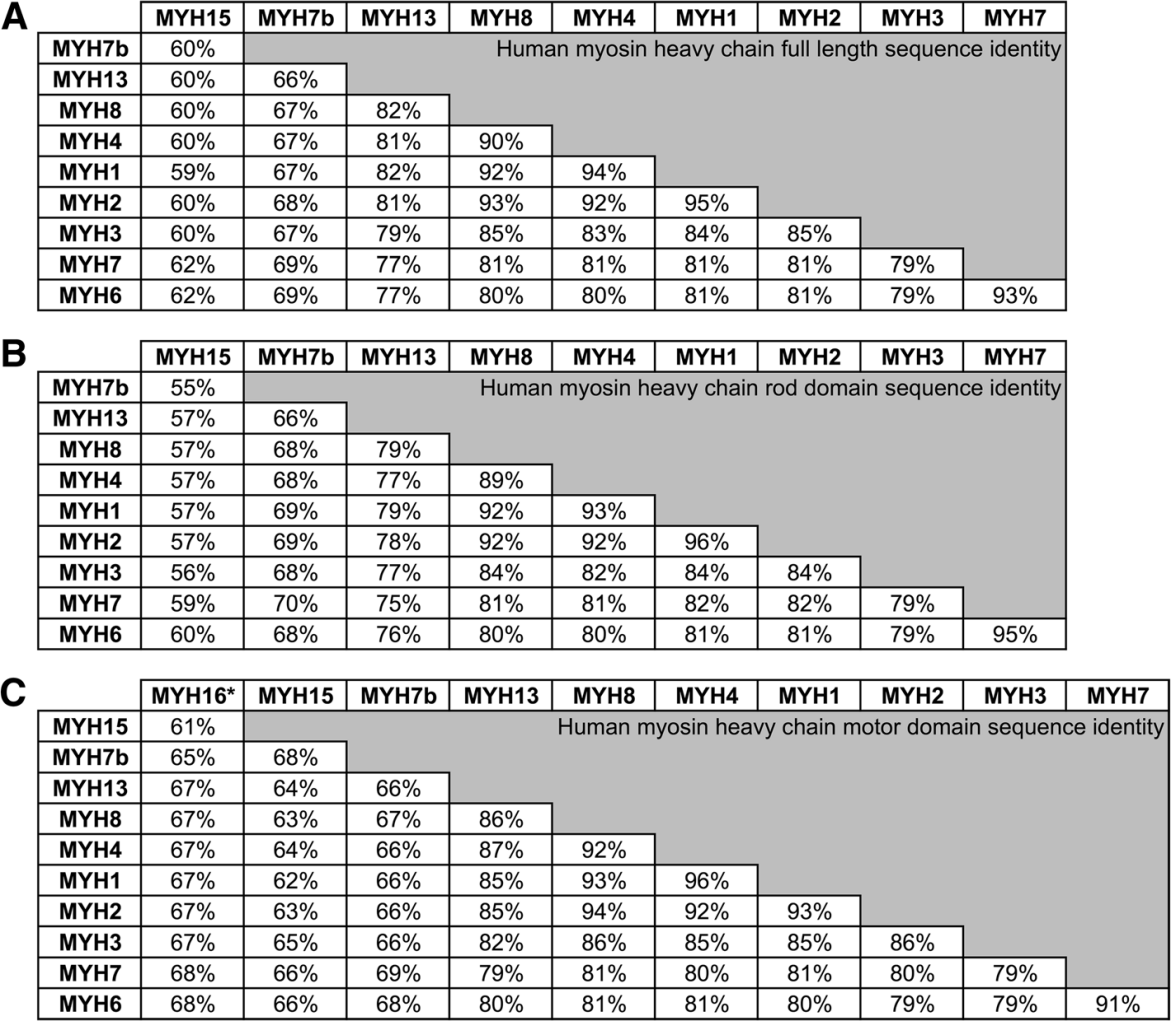
Amino acid sequence identity comparison of human sarcomeric myosin heavy chain proteins: full length sequences (**a**), rod domain sequences (**b**), and motor domain sequences (**c**). Sequences were obtained from NCBI and alignments were performed using NCBI protein BLAST. NCBI reference sequence identifiers: NP_055796.1 (MYH15), NP_065935.3 (MYH7b), NP_003793.2 (MYH13), NP_002463.2 (MYH8), NP_060003.2 (MYH4), NP_005954.3 (MYH1), NP_001093582.1 (MYH2), NP_002461.2 (MYH3), NP_000248.2 (MYH7), NP_002462.2 (MYH6). *Human MYH16 amino acid sequence was deduced by the predicted mRNA sequence (Gencode V28 Transcript Annotation ENST00000439784.7) up to the frameshift mutation in codon 660 (within the motor domain)

Vertebrates express 11 sarcomeric myosin heavy chain (MYH) genes in their striated muscles (Fig. 2); however, the expression level and pattern varies greatly across species, developmental timepoint, and muscle type [2, 3]. All 11 MYH genes are expressed in mammalian striated muscles, albeit some in extremely low abundance and some localized to just one or two specialized skeletal muscle fibers. These genes, their protein products, and muscle type of expression are listed in Fig. 2. Though humans encode *MYH4* and mRNA has been detected in extraocular [4] and jaw muscles [5], as well as in cases of Duchenne muscular dystrophy [6], this isoform is not expressed at the protein level in humans. Furthermore, *MYH16* is a pseudogene in humans and does not produce any functional protein [7]. However, other mammals express *MYH4* and *MYH16* at the protein level in fast-twitch skeletal muscle fibers (known as Type II fibers), alongside the other skeletal muscle-specific myosin isoforms [8, 9]. *MYH6* (α-MyHC) and *MYH7* (β-MyHC) are known as the cardiac myosin isoforms and are expressed in the mammalian heart. However, *MYH7* is also the dominant isoform expressed in slow-twitch skeletal muscle fibers (also known as Type I fibers), and both *MYH6* and *MYH7* are found in certain specialized skeletal muscles. Finally, recent genomic analysis identified *MYH7b* and *MYH15*, which are expressed in various specialized muscles of mammals such as the extraocular muscles and muscle spindles. Sequence identity is extremely high among the human sarcomeric myosin genes (Fig. 3), and yet, each myosin’s contractile properties, such as ATP turnover and force production, vary from isoform to isoform [10–12]. These variations in motor properties confer unique characteristics to different muscle types, and the varied myosin composition within skeletal muscle fibers allows for a wide range of contractile velocities and forces among different muscle types [11, 13]. For example, MyHC-embryonic and MyHC-perinatal are expressed in developing skeletal muscles when the demands on muscle are lower due to decreased load [2]. In adult skeletal muscle, Type II fibers express varying ratios of the adult skeletal muscle myosin isoforms, which have relatively high ATP-turnover rates and confer fast contractile properties to these fibers [10]. In contrast, β-MyHC, the major human cardiac isoform also found in slow Type I skeletal muscle fibers, is the slowest ATPase of all the human sarcomeric myosin isoforms characterized to date [11, 14]. Thus, sarcomeric myosin genes have adapted to have diverse functions, which meet the demands of various muscle types as they have evolved.

In addition to the high amino acid sequence conservation of sarcomeric myosins, the organization of the cardiac and skeletal muscle myosins in two tandemly linked genomic clusters is conserved across mammalian species [15]. In the human genome, a 350 kilobase (kb) segment on chromosome 17 contains the six skeletal muscle myosin genes (*MYH3*, *MYH2*, *MYH1*, *MYH4*, *MYH8*, *MYH13*, in order of tandem linkage) [15]. The two cardiac myosin isoforms, *MYH6* and *MYH7*, are in a second cluster on chromosome 14, separated by 4.5 kb [16]. Conversely, the three most recently discovered ancient myosins are physically unlinked to any other MYH gene in the genome; *MYH7b* is encoded on chromosome 20, *MYH15* is found on chromosome 3, and *MYH16* is on chromosome 7 [17]. The broad conservation and clustering of the MYH genes suggests two things. First, this genomic organization is important for the genes’ regulation, though they are not organized temporally [15]. Secondly, they suggest that this gene family resulted from gene duplication events of ancestral myosins [15, 17].

### Discovery of the ancient myosins

Prior to the technological advances that define the genomics age, the major sarcomeric myosins expressed in skeletal and cardiac muscle were extensively studied and characterized due to their abundance in and accessibility of these tissues. However, as the human genome was being annotated, three additional class II myosin genes were discovered: *MYH7b,*^1^ *MYH15*, and *MYH16*. In 2002, Desjardins et al. identified these three novel MYH sequences and determined their genomic locations by mapping these genes to cDNA databases. Deduced protein sequences of these genes revealed conserved sequence motifs and homology to known sarcomeric myosins, indicating that these genes are class II sarcomeric myosins [17]. The same study predicted each myosin motor’s contractile speed by comparing the sequences to characterized sarcomeric myosin motor domains; MYH7b and MYH15 were suggested to be slow isoforms whereas the protein encoded by *MYH16* (MyHC-masticatory) was predicted to be a fast isoform. However, activity assays have since demonstrated that MyHC-masticatory is more forceful rather than fast [18]. Molecular evolutionary analysis of the three novel myosins indicated that these genes are ancient and predate the well-studied skeletal and cardiac isoforms and the divergence of a smooth muscle MYH gene [17]. In fact, the ancient myosins exhibit a lower sequence identity in their full-length sequences and individual motor and rod domains to the sarcomeric class II myosins than any other sarcomeric myosin (Fig. 3), supporting the notion that they are more distantly related to the well-characterized skeletal and cardiac myosin isoforms. Since the initial discovery of the three ancient myosins, orthologs have been found in distant species including fish, chickens, snakes, and frogs, indicating that these ancient myosins were present in a common ancestor of vertebrates [19–21]. Interestingly, MYH7b and MYH15 play a prominent role in the heart and skeletal muscles of certain species like chicken and snakes, whereas in mammals, the only muscles that these myosin motors function in are highly specialized muscles (Leinwand unpublished [21]). The *MYH16* gene is expressed exclusively in muscles that originate from the first pharyngeal arch; *MYH16* is primarily expressed in the muscles of mastication but is also present in the tensor veli palatini and tensor tympani of certain species [22]. While *MYH16* expression has been observed in the jaw muscles of some vertebrates including cats [23] and crocodiles [24], a frameshift mutation led to the loss of *MYH16* expression in humans [7]. Though these ancient myosins are less studied, they are clearly set apart from the striated isoforms that have been extensively characterized and shown to play major roles in mammalian heart and skeletal muscle function. Little is known about why these myosins are absent from conventional striated tissues of mammals, unlike in reptiles and birds, and the evolutionary pressures that resulted in differential expression of these myosins across species. The three ancient myosins will be further discussed in the subsequent sections of this review.

### Evolutionary perspective of the myosin genes

In order to understand the evolutionary relationships between the myosin genes within and across species, it is important to consider a broad perspective of muscle evolution itself. Smooth and striated muscle cells are unique to specific members of the animal kingdom [25, 26]. Originally, animal striated muscles were presumed to share a common origin based solely on the ultrastructural similarity of the highly ordered striated muscle tissue across phyla [27, 28]. However, molecular phylogenetic analysis encompassing animals, fungi, plants, and protists now strongly suggests that striated muscles are a result of convergent evolution. In one study, Steinmetz and colleagues discovered a core set of muscle proteins in organisms that predate the evolution of multicellular organisms [29]. Previously, researchers hypothesized that a gene duplication event gave rise to two distinct MYH orthologs in bilaterians, accounting for the presence of both visceral smooth muscle and somatic striated muscle [30, 31]. More recent genome mining revealed that this duplication event occurred before the origin of muscle cells and that at least two distinct myosin isoforms were present in a shared common ancestor of all animals [29]. These two ancient myosin isoforms are referred to as SM-MHC and ST-MHC and have since diversified from each other with respect to assembly, contractile function, and cell type [32]. Smooth and nonmuscle myosin orthologs resulted from duplications of SM-MHC, while ST-MHC underwent duplication events to produce the current suite of sarcomeric myosin isoforms present today including *MYH7b*, *MYH15*, and *MYH16*, which were the first sarcomeric myosin genes to diverge from the ancestral myosin genes (Fig. 2) [17, 29].

**Fig. 4:**
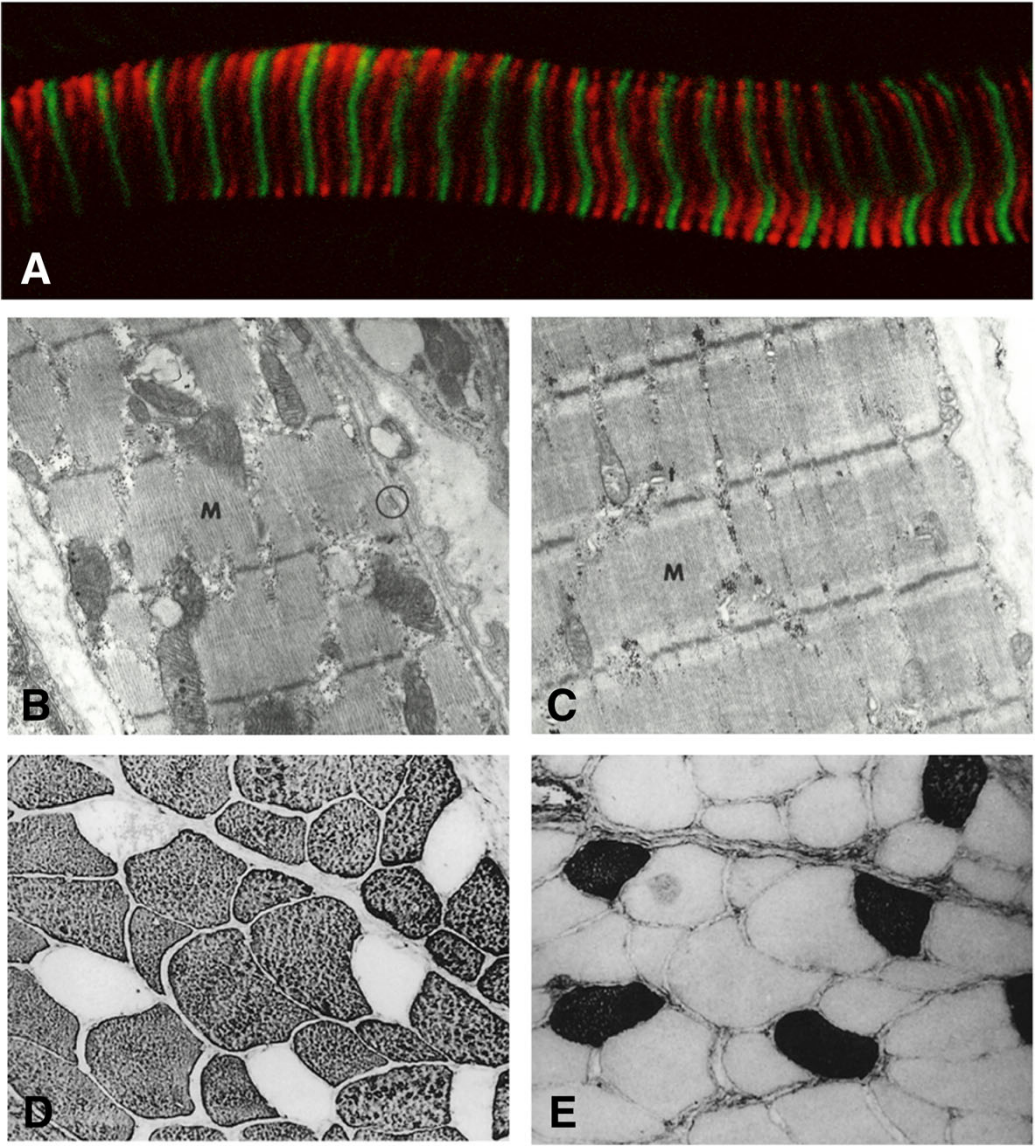
**a** Longitudinal section of rat extraocular muscle stained with MYH7b (red) and α-actinin (green) adapted with permission of the publisher from Rossi et al. 2010, copyright©2010 John Wiley and Sons. **b**, **c** Electron micrograph of muscle spindle chain fiber (**b**) characterized by clearly defined sarcomeres and large mitochondria and bag fiber (**c**) characterized by less well-defined sarcomeres and fewer mitochondria Reprinted with permission of the publisher from Ovalle et al. 1971, ©1969, CCC Republication. **d**, **e** Immunoperoxidase staining of MyHC-masticatory (**d**) and β-MyHC (**e**) in cat masseter muscle sections shows the majority of fibers are comprised of MyHC-masticatory and only a small proportion of fibers express β-MyHC, in contrast to humans, which express β-MyHC as the predominant isoform. Reprinted with permission of the publisher from Kang et al. 2010, ©2010, SAGE Publications

These three ancient myosins are expressed in mammalian specialized muscles (Fig. 4), which is logical as the majority of these tissues are thought to predate conventional striated muscles. In this review, we define specialized muscles as skeletal muscles that have adopted a unique structural organization to perform a specific function, while cardiac muscle and trunk/limb skeletal muscles are referred to as conventional muscles. Specialized muscles include extraocular muscle, muscle spindles, and masticatory muscle, which will be further discussed in this review [33]. It is worth noting that the intrinsic laryngeal muscles are also considered to be specialized muscles, but are not known to express any of the ancient myosins and are therefore outside the scope of this review. The three ancient myosins appear to have niche roles in specialized mammalian muscles, but given their diverse expression levels and variable abundance across species, several questions remain. Are these myosins required for the specific function of the specialized muscles in which they reside? Could lack of selective pressure lead to the replacement of these myosins by a more diversified set of sarcomeric myosins? Can these myosins aid our understanding of early striated muscle evolution? In this review, we will first introduce the specialized muscles and then turn our focus to *MYH7b*, *MYH15*, and *MYH16* and their roles in these specialized muscle types.

## The specialized muscles

### Extraocular muscles

Extraocular muscles (EOMs) are the muscles that surround the eyeball and control a variety of diverse and complex eye movements. These movements are necessary for eyesight precision and range from fast movements to slower pursuit and vergence movements to fixation [34]. Six EOMs control eye movement across vertebrates including four rectus muscles (medial, lateral, inferior, and superior) and two oblique muscles (superior and inferior). Each EOM originates from the posterior of the eyeball near the optic nerve and attaches to the front of the eyeball with the exception of the inferior oblique muscle [35]. The medial and lateral rectus muscles control horizontal eye movements. Vertical eye movement is achieved through the coordinated action of the superior and inferior rectus and oblique muscles, the latter of which also function in torsional movements [34–36]. Mammals possess an additional principal EOM called the levator palpebrae, which controls eyelid elevation [35]. Each EOM compartmentalizes into two layers, the thin orbital layer that lies along the EOM surface facing the bony orbital wall and the thicker global layer that faces the eyeball globe [37]. The orbital layer and global layer have distinct features as does the marginal zone, a third layer described in human EOM that lies on the orbital layer’s outer surface [38]. Within these layers, there are six distinct fiber types that participate in all eye movements, and they are classified by fiber location (global or orbital layer), innervation (singly or multiply innervated fibers), and histochemical features [34, 37].

Mammalian EOMs express an unusually high number of striated muscle myosin genes including developmental and adult isoforms as well as an EOM-specific isoform. In total, mammalian EOMs express 10 of the 11 sarcomeric myosin isoforms: the adult skeletal myosins, MyHC-IIa, MyHC-IIx/d, and MyHC-IIb; the developmental isoforms, MyHC-embryonic and MyHC-perinatal; the cardiac isoforms, β-MyHC and α-MyHC; the EOM-specific isoform, MyHC-extraocular; and two of the ancient myosins, MYH7b (Fig. 4a) and MYH15 [21, 39–44]. Interestingly, the extraocular and laryngeal muscles are the only tissues that express *MYH13*, a gene first identified by Wieczorek et al. and later discovered to evolutionarily predate the fast skeletal isoforms [45–47]. The *MYH13* protein product, MyHC-extraocular, was categorized as a fast myosin and observed in the innervation zone of both the EOM orbital and global layers, likely contributing to fast eye movements [47, 48]. Myosin isoform composition within the EOMs varies with respect to the orbital and global layers and within the muscle fiber longitudinally [41, 43, 49]. EOMs also exhibit heterogeneity in myosin composition across species. In rodents, specific MYH isoforms appear to be preferentially expressed in a certain layer [43], whereas in humans most MYH isoforms are observed in both layers [44]. Furthermore, many EOM fibers co-express multiple myosins, resulting in extremely complex expression patterns across species [43, 44, 48–50]. Based on this complex expression profile, it seems that EOM function cannot be assigned to a specific layer or set of myosin isoforms. Thus, it is likely that the comprehensive myosin expression pattern in the EOMs is critical for maintaining the demanding and diverse eye movements.

Gene expression profiling has revealed distinct differences between EOMs and other striated muscles, indicating that EOMs are highly specialized muscles. DNA microarray was used to identify differential gene expression between EOM and representative cardiac, skeletal, and smooth muscles, revealing that EOM is substantially different than other striated muscles [51]. Expression data also revealed that EOM has a distinct energy metabolism profile where genes related to glycogen metabolism are downregulated relative to skeletal muscles [51, 52]. Gene expression profiling has also identified a regulatory gene found preferentially in EOMs called the paired-like homeodomain transcription factor 2 (Pitx2) [52, 53]. Unlike in conventional skeletal muscles, Pitx2 is required for EOM differentiation and development in mice and appears to regulate gene expression related to MYH isoforms, contractility, and EOM fiber size [53–56]. Pitx2 is also highly expressed in a population of EOM myogenic precursor cells in healthy and muscular dystrophy mice, suggesting a role for Pitx2 in the EOM’s regenerative capabilities that may contribute to preferential EOM sparing in certain diseases [57].

One yet unresolved question in the field pertains to why EOM is preferentially involved in or spared in certain diseases. The EOMs are preferentially impacted in myasthenia gravis, an autoimmune disease that targets acetylcholine receptors of the neuromuscular junction. This is hypothesized to be attributable to the differences in the number and isoforms of acetylcholine receptors and innervation properties at the neuromuscular junction of EOM versus typical skeletal muscles [34, 58]. Likewise, differences in the immune response between EOMs and skeletal muscle likely underlie the selective involvement of the EOM in the autoimmune disease Graves’ ophthalmopathy [59, 60]. Conversely, despite the widespread muscle degeneration that characterizes muscular dystrophy, the EOMs are spared in this disease and patients retain normal EOM function [61]. Expression profiling revealed that there are no clear changes in gene expression in EOMs of a muscular dystrophy mouse model, indicating that EOM remains unaffected in the disease state. Thus, the characteristics that distinguish EOM from conventional skeletal muscles may be protective in this disease [62, 63]. A current hypothesis in the field predicts that a population of myogenic precursor cells mediates the EOM’s regenerative capacity and contributes to EOM sparing in muscular dystrophy [64]. In fact, EOMs of aged and muscular dystrophy mice retain an enriched population of muscle precursor cells with proliferative capacity compared to limb skeletal muscle [64, 65]. Though the precise mechanism remains undefined, the EOM’s susceptibility and protection from certain diseases likely stems from a combination of its unique features, including distinct myosin isoform expression patterns, fiber types, gene expression profile, and developmental requirements.

### Muscle spindles

Muscle spindles are the innervated sensory structures buried within conventional striated muscles that relay information about muscle length and stretch to the central nervous system [66–68]. Muscle spindles provide essential sensory input about the spatial placement, extension, and contraction rate of muscles to the central nervous system, which is required for effective voluntary control of striated muscles [66, 69]. They provide a static and a dynamic stretch response by detecting both the initiation and the continuation of stretch, respectively [66, 69, 70]. Muscle spindles are differentially distributed throughout the body’s muscles and are typically focused along axial regions and in smaller muscles, though the reason for this distribution is unclear [71, 72]. Mice deficient in Egr3, a transcription factor essential for muscle spindle development, have severe phenotypes including loss of voluntary muscle control, scoliosis, resting tremors, and eyelid sagging, even though the extrafusal muscle fibers are normal [69]. Thus, muscle spindles are necessary for normal locomotion.

The general muscle spindle morphology consists of a bundle of intrafusal muscle fibers that are separated from the extrafusal fibers of the surrounding skeletal muscle [66]. There are three types of intrafusal muscle fibers: bag_1_, bag_2_, and chain fibers [66–68, 73]. Bag and chain type fibers are distinguished by their nuclear distribution within the muscle fiber; bag fibers have centrally located nuclei, whereas chain fiber nuclei are equally distributed along their length [67]. In addition, electron microscopy studies have shown distinct sarcomeric, myofibril, and mitochondrial organization between these two muscle spindle fiber types. Chain fibers have clearly defined myofibril units and numerous, large mitochondria that span one to two sarcomeres each (Fig. 4b) [74, 75]. The organization in bag fibers is less clear, with less well-defined sarcomeres that are tightly packed and have fewer, smaller mitochondria than chain-type fibers (Fig. 4c) [74, 75]. Bag_1_ fibers are distinguished from bag_2_ fibers by a lower ATPase activity and contain a larger proportion of slow myosin isoforms such as β-MyHC [66, 76]. Functionally, bag_1_ fibers are solely responsible for the dynamic stretch response, whereas both bag_2_ and chain fibers contribute to the static stretch response [66, 67]. Stretch information, and therefore proprioception, is passed through the afferent fibers that innervate the muscle spindles to the central nervous system [66, 67].

The muscle spindle acts as the primary stretch receptor of the muscle to relay feedback information to the nervous system, which in turn regulates the length and tone of the extrafusal fibers. The myosin-actin crossbridge is essential to this muscle spindle function. The initial stretch response recorded by the spindle is thought to be the result of breaking static crossbridges by stretching the muscle [66, 67, 77]. This causes changes in spindle stiffness, triggering mechanosensitive ion channels to initiate a signal cascade [67, 77]. The crossbridge reformation rate also creates signals downstream of the original stretch, which influences whether or not the muscle remains extended [66]. Myosin composition therefore plays an important role in muscle spindle response rate. There is a wide range of sarcomeric myosins expressed in muscle spindles across species, including β-MyHC, α-MyHC, MyHC-embryonic, MyHC-perinatal, and MyHC-IIa [76]. Two of the ancient myosins, MYH7b and MYH15, have also been found in muscle spindles [21]. Keeping in mind that bag_1_ and bag_2_ fibers are differentiated by their variable ATPase content, it is probable that the myosin expression profile of each intrafusal muscle fiber is an important determinant of its activity [66]. The need to finely tune the crossbridge dynamics of different muscle spindles present in the body could explain why the two ancient myosins, *MYH7b* and *MYH15*, are expressed in muscle spindles. It is possible that the expression levels of these ancient myosins contribute to the precise contractile needs of muscle spindles in order to garner a robust stretch response in different muscles.

### Masticatory muscles

The masticatory muscles move the jaw to accomplish a wide range of activities including swallowing, chewing, speaking, and infantile suckling. The diverse functions of the jaw muscles are in part due to their unique structure, which allows the muscle to exert precise control over the mandible (i.e., detached lower jaw bone). In humans, four muscles comprise the masticatory apparatus: the masseter, temporalis, medial pterygoid, and lateral pterygoid. The first three muscles function to elevate the jaw, while the lateral pterygoid is a jaw-opening muscle [78]. The masseter muscle is the most superficial masticatory muscle, originating from the zygomatic arch (i.e., cheekbone) and attaching to the mandible. The masseter is the most powerful of the four muscles allowing for forceful jaw closures. The temporalis muscle originates in a shallow depression on the side of the skull known as the temporal fossa and converges into a tendon that attaches to the mandible. The temporalis muscle can retrude the jaw as well as close the jaw. The last of the jaw-closing muscles is the medial pterygoid. Both the medial pterygoid and lateral pterygoid are attached to the inner surface of the jaw and can protrude the mandible. The lateral pterygoid is responsible for moving the jaw laterally and is also involved in protruding and depressing the mandible [78].

The masticatory unit in humans has evolved to be a highly specialized muscle group. The complex muscle fiber architecture, heterogeneity, and myosin composition of the masticatory muscles allow for a wide range of forces and contractile velocities. The mastication muscles are made up of a variable population of pure and hybrid muscle fibers. Pure fibers expressing a single myosin isoform are found for slow β-MyHC, MyHC-IIa, and MyHC-IIx/d. Hybrid fibers expressing a combination of these isoforms, as well as MyHC-embryonic and α-MyHC are also present [79]. Immunohistochemistry studies on male and female human cadavers demonstrated that all four masticatory muscles express approximately 70% β-MyHC. The temporalis and masseter muscles show a relatively equal distribution of the remaining MyHCs present, while the pterygoids show a marked increase in MyHC-IIa and α-MyHC compared to MyHC-IIx/d and MyHC-embryonic [80]. *MYH4* transcripts are abundantly expressed without accompanying MyHC-IIb protein expression [5], similar to previous reports of *MYH4* expression in other human skeletal muscles [6, 8]. The fiber-type population within the four jaw muscles varies greatly, as does fiber size itself [80, 81]. Interestingly, differences in fiber composition between males and females have been observed in the masseter muscle of rabbits [82] and mice [83]. In humans, a significant increase in the cross-sectional area (CSA) of masseter Type I and Type II fibers has been observed in males compared to females [84–86]. Hence, the masseter muscle appears to be sexually dimorphic in certain species of mammals. Diverse expression of specific myosin motors in the masticatory muscles allows for energy consumption at the cellular and animal level to be fine-tuned via the non-redundant ATPase properties inherent to different isoforms [87]. This complex sarcomeric myosin expression pattern suggests that the masticatory muscle is highly adaptable. Indeed, changes in fiber type, CSA, and the contractile mechanics of jaw muscles have been observed in response to diet [88–97], aging [98–105], and craniomandibular disorders [85].

Alterations in craniofacial morphology have been correlated with the composition and function of temporalis and masseter muscles, but it is unclear whether the jaw muscles are the driving force behind craniofacial morphological alterations or if the muscle function adapts to changes in external stimuli from the skeleton. There are three basic facial forms categorized for humans: long, average, and short [106]. There is greater facial morphology variation in individuals with weak jaw muscles [107], while stronger and thicker jaw muscles produce more uniform facial morphological features suggesting that if the masticatory muscle is too weak, it exerts less influence over facial morphology [108]. The jaw muscles are hypothesized to determine facial dimensions of humans [109]. The jaw-closing muscles of short-faced individuals have thicker fibers [109, 110], while those of long-faced individuals have thinner fibers [111, 112], and produce lower molar bite forces [113–115]. Differences in fiber type are also evident in people with different facial forms. Rowlerson et al. observed that patients with an open bite presented with a higher proportion of slow Type I fibers, while patients affected by deep bite presented with a higher proportion of fast Type II fibers [116]. Boyd et al. observed that a longer face correlated with more Type II fibers in masseter muscles [117]. In addition, several animal studies indicate that the jaw muscles may play a role in skull growth and development (for review see Kiliaridis [108]). Surgical manipulation of the temporalis muscle in young rabbits directly resulted in changes to the local skull morphology and cranial development, although this was not correlated with any specific myosin isoform [118]. A similar study in primates revealed that altering the temporalis and masseter muscle attachments could alter craniofacial growth patterns [119]. Thus, the masticatory muscles are a complex unit that attach to the skull and jaw and can influence the phenotypic characteristics of the cranium.

The jaw made its first appearance in the evolutionary timeline over 400 million years ago when gnathostomes, or jawed vertebrates, first diverged [120]. It is thought that the jaw evolved through adaption of ancient gill cartilages [121]. The predatory success of early gnathostomes is largely attributed to the presence of the jaw [122], and today, gnathostomes make up 99% of vertebrate species. With the radiation of vertebrate and mammalian speciation, the jaw has had ample time and opportunity to evolve to the functional needs of a wide range of animals. The *MYH16* gene is the oldest of the sarcomeric myosins present in vertebrates and is primarily expressed in jaw muscles. MyHC masticatory, which is associated with a high contraction force, has been observed in certain carnivorous species, such as sharks and cats (Fig. 4d, e) [123], where forceful jaw closures would be advantageous for predation. The jaw muscles have evolved to serve functions that are distinct from conventional skeletal muscle (locomotion and postural tonicity). By expressing a wide variety of myosins, masticatory muscle is able to regulate the force, speed, and energy efficiency of contraction in order to adapt to the ever-changing environments and demands of various animals.

## The ancient myosins

### MYH7b

Human *MYH7b* is a 27-kb gene found on chromosome 20. *MYH7b* shares the highest sequence identity to *MYH7* (β-MyHC) and *MYH6* (α-MyHC), reaching 69% sequence identity at the amino acid level (Fig. 3). In addition, each of these three myosin genes harbors an intronic microRNA; *MYH6* encodes miR-208a in intron 27, *MYH7* encodes miR-208b in intron 31, and *MYH7b* encodes miR-499 in intron 19 [124, 125]. miR-208a is cardiac myocyte-specific and has known roles in cardiac stress response regulation, whereas miR-208b and miR-499 play regulatory roles in the heart and have redundant roles in skeletal muscle fiber-type specification [124, 125]. Though *MYH7b* shares a high sequence homology and features with the two human cardiac myosin isoforms, this myosin’s expression pattern and regulation are unique in mammals.

*MYH7b* was first identified by Nagase et al. in an effort to categorize previously unknown human genes [126]. The *MYH7b* sequence (designated KIAA1512) was cloned from a human fetal brain cDNA library and mapped to chromosome 20. Expression profiling by the authors revealed that *MYH7b* RNA is highly expressed in the heart, skeletal muscle, adult, and fetal brain and more lowly expressed in the ovary, kidney, lung, liver, pancreas, and spleen [126]. As the human genome was being annotated, Desjardins et al. identified *MYH7b* as one of three ancient myosins belonging to the sarcomeric myosin family of genes [17]. *MYH7b* transcripts were confirmed in several conventional mammalian muscles including the heart, soleus, tibialis anterior, quadriceps, and diaphragm [21, 127]. A 2012 study by Warkman et al. reported the presence of MYH7b protein in the mouse heart; however, this finding was later retracted as this result was due to non-specific antibody reactivity [128]. To date, MYH7b has not been detected at the protein level in mammalian cardiac or skeletal muscle. This discrepancy between *MYH7b* RNA and protein expression in conventional muscles is due to a non-productive splicing event in which the transcript is produced, but undergoes some nonsense mediated decay, while miR-499 expression is retained in these tissues [127]. Despite this expression pattern in the heart and skeletal muscle, MYH7b protein is present in specialized muscles of mammals including the EOMs and muscle spindles. In rats, MYH7b was detected in EOM orbital and global layers (Fig. 4**a**) and observed in varying abundance in muscle spindle bag_1_ and bag_2_ fibers [21]. Both of these specialized muscles express many sarcomeric myosins and carry out highly specific tasks. Thus, *MYH7b* may have a role that uniquely fits a contractile requirement of these muscles.

*MYH7b* transcripts capable of encoding protein have also been observed in the mouse brain [127]. Corroborating this finding, Rubio et al. identified *MYH7b* in a yeast two-hybrid screen designed to identify actin regulators in the rat brain. Subsequently, this group used siRNA to target *MYH7b* in cultured hippocampal neurons and observed alterations in dendritic spine morphology and excitatory synapse strength, indicating that *MYH7b* contributes to dendrite structure and function [129]. *MYH7b* is also implicated in several diseases that involve both muscle and nonmuscle phenotypes. In 2014, Haraksingh et al. reported that compound heterozygous mutations in *MYH7b* are associated with sensorineural hearing loss. Exome sequencing on a family with sensorineural hearing loss in three children revealed two mutations in *MYH7b*: a maternally inherited mutation in the *MYH7b* motor domain and a paternally inherited mutation in the *MYH7b* rod domain [130]. These are the only known associations between *MYH7b* and deafness, and thus is an unusual occurrence. Whole exome sequencing has also identified missense mutations in *MYH7b* associated with left ventricular non-compaction cardiomyopathy [131]. Recently, a genome-wide association study (GWAS) identified associations between congenital heart defects involving left-sided lesions and chromosome 20q11, a region that includes *MYH7b* [132]. Another GWAS aiming to identify new cutaneous melanoma risk loci identified significant associations with 20q11 and *MYH7b* [133]. However, the variants associated with *MYH7b* are hypothesized to be positional markers for genes associated with melanoma-like *ASIP*, a pigmentation gene in the same chromosomal region as *MYH7b*, rather than a disease modifying variant [134, 135].

Though MYH7b does not appear to play a major role at the protein level in conventional muscles of mammals, MYH7b orthologs are found in fish, chickens, snakes, and frogs and appear to have predominant roles in the conventional muscles of these species. In chickens, the MYH7b ortholog is expressed in the Purkinje fibers of the heart, as well as slower skeletal muscles such as the anterior latissimus dorsi (Leinwand unpublished [21, 136]). The fish genome also contains *MYH7b* orthologs, and it appears that gene duplication has resulted in multiple *MYH7b* paralogs [19, 20]. For example, *torafugu* have two gene duplicates of the *MYH7b* ortholog; one is specifically expressed in slow skeletal muscle whereas the other has broader expression in adult and embryonic tissue [20]. Syntenic organization of *MYH7b* is consistent in Xenopus, chickens, mice, and humans, further validating the concept that this myosin is derived from an ancient myosin found in an ancestor common to these species [21, 137].

### MYH15

*MYH15* is encoded on human chromosome 3. Its genomic sequence is unusually large, around 142 kb, and genomic analysis led to the conclusion that it is one of three ancient myosins found outside of the canonical sarcomeric myosin clusters [17]. Since the identification of *MYH15* in humans, orthologs have been found in chickens, snakes, and various mammals, though no such ortholog has been found in teleost fish [20, 138]. The *MYH15* ortholog found in chickens is the major myosin found in the adult ventricle and is also important for embryonic development (Leinwand unpublished [138]). These data suggest that MYH15 acts as a conventional sarcomeric myosin. However, the expression pattern of *MYH15* in mammals does not follow that of a typical sarcomeric myosin. Instead, *MYH15* transcripts have been observed in several brain regions, the testis, and the pituitary gland [139, 140]. *MYH15* RNA has also been described as a long non-coding RNA found in the ovaries of sheep and is suggested to be important for fecundity in these animals [141, 142]. MYH15 protein has been identified in mammalian muscle tissue via immunostaining in rat EOMs and muscle spindles [21]. However, in humans, MYH15 protein has only been observed in the pulmonary vascular epithelium and alveolar macrophages [143]. This unusual expression pattern in mammals suggests a role for MYH15 outside of muscle.

The role of MYH15 in muscle spindles is unclear. Muscle spindle myosin expression profiles vary regionally throughout the spindle, and isoform variation influences the myosin-actin crossbridge dynamics known to be important for the response of the muscle spindle [66, 68, 76, 77]. Thus, the predicted slow ATPase rate of *MYH*15 may contribute in some way to the specificity of muscle spindle signaling. There are further clues for this myosin’s function in the pulmonary vascular system, as disease-associated single-nucleotide polymorphisms (SNPs) in *MYH15* correlate with noncardioembolic stroke, coronary heart disease, and chronic obstructive pulmonary disease in humans [143–147]. In addition, a SNP in *MYH15* has been linked to bovine pulmonary hypertension, supporting a role for *MYH15* in the pulmonary system [148]. The associated diseases in humans are all caused by obstructions in the vascular system, which suggests that MYH15 may have a role in clearing such blockages. Even though *MYH15* shares the heptad repeat rod motif universal to sarcomeric myosins and is the major ventricular MyHC in chicken hearts, the identified expression pattern in mammals suggests the potential for a role outside of the sarcomere [17]. Given that ancestral myosins were present before the evolution of a muscle cell, it is reasonable to contemplate that the first myosins to diverge (i.e., *MYH15*) could retain a contractile or motile role outside of a muscle cell.

### MYH16

Though the human *MYH16* gene is a pseudogene, it has 42 predicted exons and encompasses 68 kb, spanning three times the physical size of a typical sarcomeric myosin [7]. Phylogenetic analyses revealed that *MYH16* predates all other vertebrate MYH isoforms and is the most distantly related MYH gene compared to all others in vertebrates [17, 149]. The human *MYH16* sequence has five additional introns that are only shared with the striated muscle gene of mollusks, further supporting the notion that *MYH16* is one of the most ancient myosins in the animal kingdom [17]. Zhu et al. showed that *MYH16* and its syntenic region are not present in the mouse genome, indicating *MYH16* was lost in mice due to a genomic deletion [150]. In fact, the *MYH16* gene has been independently lost from several other mammalian lineages [22, 151, 152], indicative of the susceptibility of jaw-closing muscles to selective pressures [9]. A trend of *MYH16* loss is seen in mammals that typically favor faster contractions over forceful jaw contractions, which is often true for smaller animals. *MYH16* produces protein in many other vertebrate species where it is expressed in masticatory muscle. More specifically, *MYH16* orthologs have been found in the masseter and temporalis muscles of certain primates [22, 151], carnivores [9, 22–24], marsupials [9], rodents [153], bats [151], crocodiles [24], and sharks [9].

Human *MYH16* became a pseudogene due to a two-nucleotide frameshift deletion in exon 18 that results in a premature stop codon [7]. Stedman et al. predicted the *MYH16* mutation occurred 2.4 million years ago (MYA), under the assumption that the neutral mutation rate remained constant since the human-chimp divergence (6–7 MYA) [154]. According to this timeline, the genetic inactivating mutation is predicted to precede the reduction in mass of the mandible in the human ancestor, *Homo erectus*, which occurred around 2.0 MYA [155] simultaneously with a shift toward a larger cranium [156]. This led many to hypothesize that the loss of *MYH16* in early *Homo* resulted in small jaw muscles and less tension applied to the cranium during development, allowing for increased cranial capacity and encephalization. Mechanical measurements of pure MyHC-masticatory fibers isolated from the masseter and temporalis muscles of dogs and cats showed that MyHC-masticatory was the most forceful of all the myosin proteins [18, 157]. Contrary to the prediction of MyHC-masticatory constituting a “superfast” myosin [17], one study showed that MyHC-masticatory fibers had an intermediate contraction velocity, most similar to that of MyHC-IIa [18]. The notion that loss of *MYH16* corresponded to the loss of the most forceful myosin in the human jaw has lent itself to the hypothesis that encephalization resulted from reduced tension in the jaw. A more comprehensive analysis of *MYH16* evolutionary history later called into question the proposed date of the *MYH16* mutation, suggesting the gene may have been inactivated 5.3 MYA, which precedes the first appearance of *Homo* and the modern day cranium [158]. In 2015, the same authors revisited this question by analyzing nuclear genomic sequences of multiple hominin lineages and concluded that the inactivating mutation occurred before the Human-Neanderthal divergence 0.6 MYA (as they share this mutation) [159], and after the human-chimpanzee divergence, consistent with the 2.4 MYA estimate by Stedman et al. [160].

Despite this timeline, some have argued that *MYH16* inactivation would not contribute to an increased cranial capacity, mainly because the majority of brain growth precedes the formation of the masticatory apparatus during fetal development [161]. Other researchers have instead proposed that smaller jaws were the cause of the mutation. A dietary shift to consuming softer foods could have resulted in smaller jaw muscles, leading to a lower dependency on the *MYH16* isoform. The lack of selective pressure could have in turn resulted in the loss of *MYH16* [9, 160]. Nevertheless, the loss of *MYH16* in humans has been proposed to be associated with a marked reduction in masticatory muscle mass, which could have allowed for human encephalization [7, 162]. While loss of the MyHC-masticatory in humans might not have directly resulted in encephalization, it is a fascinating example of how genetic inactivation can lead to the acquisition of human-specific phenotypes [163].

## Conclusions

The discovery of the three ancient myosins, *MYH7b*, *MYH15*, and *MYH16*, completed the inventory of the mammalian sarcomeric myosin genes. For decades, these myosins escaped detection due to their limited expression in the specialized tissues of mammals including the EOMs, muscle spindles, and the masticatory muscles. Even 17 years after their annotation in human genome, many questions about the ancient myosins and their role in specialized muscles remain unanswered. One such question is whether the ancient myosins have an essential role in specialized muscles or whether they are being eliminated through evolutionary processes in mammals. Take, for example, one of the characterized sarcomeric myosins, *MYH13* (MyHC-extraocular), that is expressed in two highly specialized muscles (EOM and laryngeal muscle). Given that MyHC-extraocular has the fastest contractile rate of the sarcomeric myosins and is expressed exclusively in muscles that require superfast contractions (e.g., eye movements and phonation), it appears that this myosin has evolved to fulfill unique roles in these specialized muscles. Therefore, the argument can be made that *MYH13* expression is required for proper muscle function in specialized muscles. Likewise, *MYH16* may too be required for the proper function of the specialized masticatory muscles in certain species given its exclusivity in this tissue type and its high force-generating capability compared to the other sarcomeric myosins. A different argument could be made for MYH7b and MYH15 function in the EOM and muscle spindles. These specialized muscles are known to contain multiple myosin isoforms, which is hypothesized to allow the muscle to be functionally adaptable. In contrast to the idea that each myosin performs a specific task, perhaps this varied expression of myosins, including the ancient ones, is the basis for their plasticity and adaptability wherein there is no reliance on one isoform for a specific function. Lastly, certain species may selectively express the ancient myosins due to muscle type and demand, as is seen with the well-characterized myosins. Preferential expression of these ancient myosins at the protein level in conventional muscle of more distantly related species (e.g., snakes and birds that express MYH7b and MYH15 protein in their cardiac and skeletal muscle) contrasts with that of mammals in which these myosin motors are restricted to specialized muscles. The majority of studies encompassing the ancient myosins are done in mammals, but there may be more to be learned from studying these isoforms in diverse species. Doing so may help answer the question of whether these myosins require niche roles to remain evolutionarily relevant. Finally, a major question remains as to whether vertebrates will continue to evolve more functionally distinct myosins to satisfy the ever-changing needs of the muscle and whether the functional repertoire of myosins will grow or if certain myosin isoforms will become obsolete in the future.

## Abbreviations

CSA: Cross-sectional area
EOM: Extraocular muscle
MYA: Million years ago
MYH: Myosin heavy chain (gene)
MyHC: Myosin heavy chain (protein)
Pitx2: Paired-like homeodomain transcription factor 2
SNP: Single-nucleotide polymorphisms

## Footnotes

1 The nomenclature at the time designated this myosin isoform as *MYH14*; however, current nomenclature now refers to this myosin as *MYH7b* to avoid confusion with the gene encoding nonmuscle myosin IIc.
